# First use of ^18^F-labeled ML-10 PET to assess apoptosis change in a newly diagnosed glioblastoma multiforme patient before and early after therapy

**DOI:** 10.1002/brb3.217

**Published:** 2014-02-12

**Authors:** Matthew J Oborski, Charles M Laymon, Frank S Lieberman, Jan Drappatz, Ronald L Hamilton, James M Mountz

**Affiliations:** 1 Department of Bioengineering, University of PittsburghPittsburgh, Pennsylvania; 2 Department of Radiology, University of PittsburghPittsburgh, Pennsylvania; 3 Department of Neurology and Department of Medicine, Division of Hematology/Oncology, University of Pittsburgh School of MedicinePittsburgh, Pennsylvania; 4 Department of Pathology, Division of Neuropathology, University of PittsburghPittsburgh, Pennsylvania

**Keywords:** ^18^F-ML-10, early-therapy response assessment, glioblastoma multiforme, positron emission tomography

## Abstract

**Objectives:**

The authors present the first use of the novel positron emission tomography (PET) apoptosis tracer ^18^F-labeled 2-(5-fluoro-pentyl)-2-methyl-malonic acid (^18^F-ML-10) for early-therapy response assessment of a newly diagnosed glioblastoma multiforme (GBM) patient.

**Case report:**

A 71-year-old male with a newly diagnosed GBM received ^18^F-ML-10 PET scans prior to therapy initiation (baseline) and after completing 3 weeks of whole-brain radiation therapy with concomitant temozolomide chemotherapy (early-therapy assessment, ETA). The baseline ^18^F-ML-10 PET scan showed increased tracer uptake at the site of the GBM, with highest activity toward the central portion of the tumor. At the ETA time point, a new distribution of tracer uptake was observed compared to baseline. Normalized pixel-by-pixel subtraction of baseline from ETA was used to quantify change in tracer distribution between ^18^F-ML-10 PET imaging time points. Results of this analysis showed reduction in ^18^F-ML-10 uptake at the site of greatest baseline uptake, but increased uptake around the periphery of the tumor at the early-therapy time point.

**Conclusion:**

The changing patterns of ^18^F-ML-10 uptake between baseline and ETA are suggestive for therapy-induced tumor cellular apoptosis.

## Introduction

Current radiologic methods for evaluating response to therapy in glioblastoma multiforme (GBM) rely largely on magnetic resonance imaging (MRI) and consist of assessing changes in tumor morphology and the degree and extent of contrast enhancement (e.g., Macdonald criteria) (Wen et al. 2010). However, the utility of such methodology is limited, as changes in tumor size can be slow relative to the timescale of the underlying molecular physiology. Moreover, the degree of contrast enhancement by a GBM can be influenced by several nontumor processes including radiation necrosis (Hygino da Cruz et al. 2011).

In tumors, the rate of spontaneous apoptosis is increased compared to normal tissue, and is often associated with tumor cell turnover (Meggiato et al. 2000). High baseline apoptotic indices in untreated tumors have been associated with both undifferentiated malignancies and lower survival rates (Meggiato et al. 2000). However, in tumors treated with effective cancer therapies (e.g., temozolomide chemotherapy, radiosurgery) size reduction has been associated with apoptosis (Witham et al. 2005; Fernandez-Luna 2007).

Therefore, given the role of apoptosis in therapeutic response of tumors, serial assessment of tumor apoptotic state through in vivo positron emission tomography (PET) imaging is highly desirable (Blankenberg 2008). A promising class of tracers proposed for molecular imaging of apoptosis is a family of small molecules developed by Aposense Ltd. (Petach-Tikva, Israel), of which the PET ligand ^18^F-ML-10 is a member (Reshef et al. 2007). In vitro studies using tritiated ML-10 (^3^H-ML-10) have shown that ^3^H-ML-10 selectively targets cells undergoing apoptosis and is not taken up by necrotic cells (Cohen et al. 2009). As a PET tracer, ^18^F-ML-10 shows a desirable rapid clearance from blood through the kidneys, and exhibits high stability in vivo (Hoglund et al. 2011).

## Case Report

### History and examination

**Figure 1:**
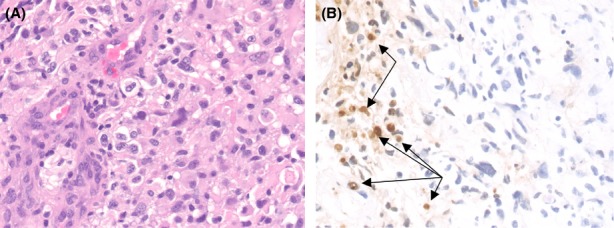
Neuropathology results for 71-year-old male patient with high-grade glioma show increased cellularity, pleomorphic nuclei, and endothelial proliferation (A, original magnification 400×) with focal areas of necrosis characteristic of a GBM. Terminal deoxynucleotidyl transferase dUTP nick end labeling in situ hybridization (B) shows scattered positive nuclei within the tumor often associated with necrotic areas, however, other areas of the tumor not associated with necrosis also showed apoptosis (arrows, original magnification 400×).

After signing informed consent documents, a 71-year-old male with a newly diagnosed GBM, confirmed by fine-needle MRI-guided stereotactic biopsy, was enrolled in a University of Pittsburgh Institutional Review Board-approved ^18^F-ML-10 apoptosis imaging protocol. Neuropathology evaluation revealed a high-grade glioma with increased cellularity, pleomorphic nuclei, and endothelial proliferation (Fig. 1A, original magnification 400×) with focal areas of necrosis characteristic of a GBM. Ki-67 immunostain showed 15% proliferation rate. Molecular studies showed deletions of 10q (PTEN gene) and 9p (p16/CDKN2A gene), both of which are seen in most GBMs. There was no epidermal growth factor receptor amplification or evidence of a p53 mutation. O(6)-Methylguanine-DNA methyltransferase-promoter methylation was not detected. Terminal deoxynucleotidyl transferase dUTP nick end labeling in situ hybridization to detect fragmented DNA associated with apoptosis showed scattered positive nuclei within the tumor often associated with necrotic areas; however, other areas of the tumor not associated with necrosis also showed apoptosis (Fig. 1B, arrows, original magnification 400×). Therapy for this subject included external beam radiation (RT) in 2 Gy fractions with concomitant temozolomide (75 mg/m^2^ daily) chemotherapy.

### Medical imaging protocol

The subject's imaging protocol included T1-MRI (Siemens 3T Magnatom Trio; Siemens, Munich, Germany) and PET (Siemens ECAT HR+; CTI/Siemens, Knoxville, TN). Imaging was performed at two time-points: baseline (prior to therapy initiation) and early-therapy assessment (ETA, 3-weeks after therapy initiation). PET scans were performed and reconstructed identically at the two time points. Each scan consisted of a 30-min acquisition performed over the range 120–150 min following intravenous (IV) injection of 10 mCi of ^18^F-ML-10. PET images were normalized to the maximum voxel value within a defined region of the superior sagittal sinus. To enable voxelwise comparison, the ETA T1-MRI was registered to the baseline T1-MRI, and each PET scan was then registered to its associated coregistered MRI scan. All image registration was performed using MIM 5.4 image analysis software (MIM Software Inc., Cleveland, OH 44122).

### Findings

**Figure 2:**
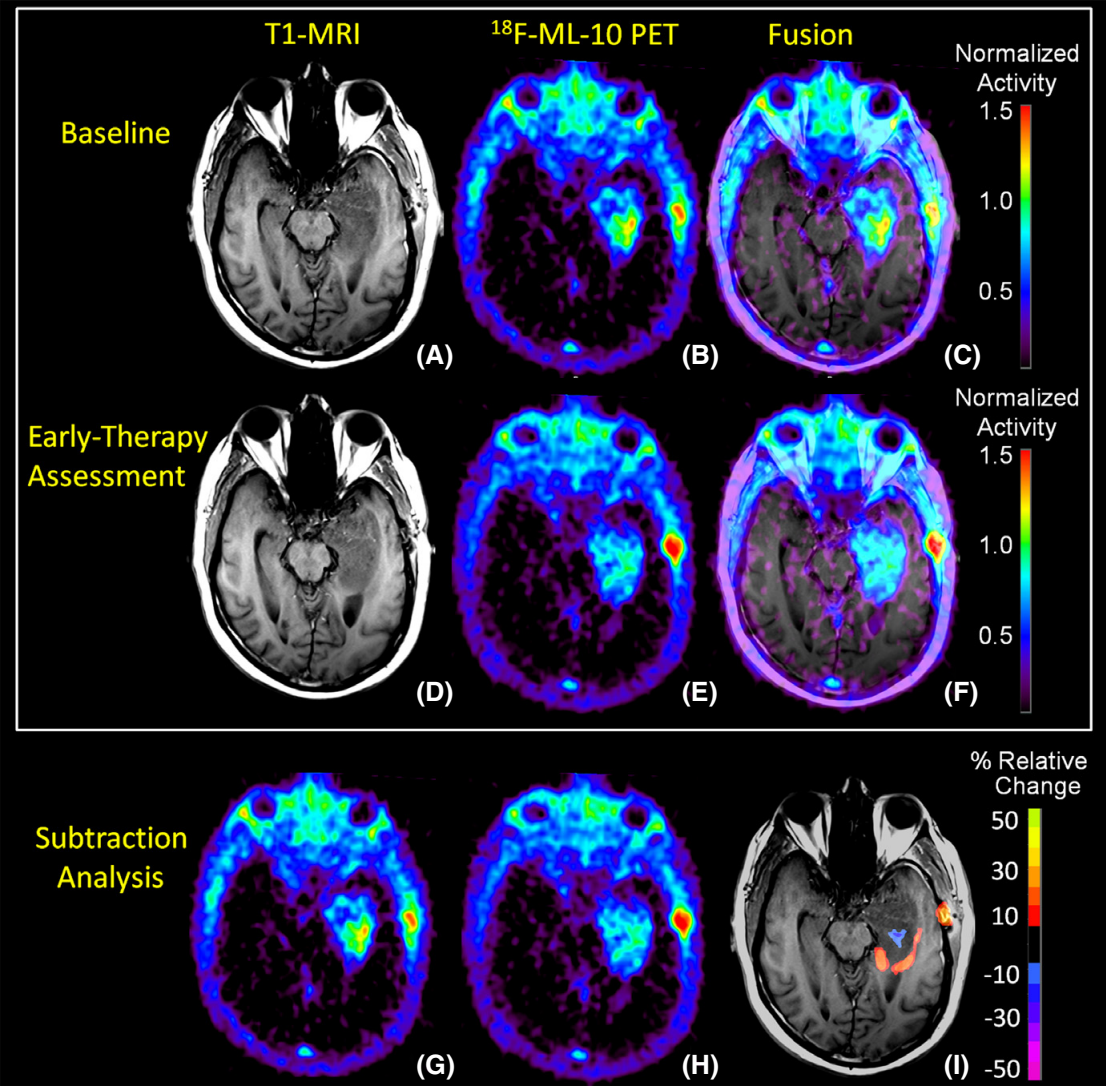
Representative T1-MRI and ^18^F-ML-10 PET imaging sections at baseline and early-therapy assessment (ETA) time-points. At baseline, the subject's T1-MRI (A) shows left temporal lobe GBM. ^18^F-ML-10 uptake at baseline PET (B) shows a region of high tracer uptake corresponding to the site of the GBM on the subject's baseline T1-MRI as seen in the PET-MRI fusion image (C). After 3-weeks of therapy (RT+temozolomide), the subject received a new set of T1-MRI (D) and ^18^F-ML-10 PET (E) images to assess for response. (F) shows the ETA T1-MRI and ^18^F-ML-10 PET fusion image. Normalized voxel-by-voxel subtraction cluster map of baseline (B and G) from ETA PET (E and H) is shown fused to ETA T1-MRI. Regions of the GBM exhibiting high baseline ^18^F-ML-10 uptake show reduced uptake at ETA (blue), while new regions (compared to baseline) of ^18^F-ML-10 uptake are observed at the tumor periphery (red/orange).

Figure 2A shows axial sections of the baseline T1-MRI scan showing the subject's GBM located in the left temporal lobe. The baseline PET image (Fig. 2B) shows a region of high tracer uptake in the tumor center with comparatively lower uptake observed on the tumor periphery. Additionally, low tracer uptake is observed in the uninvolved normal brain tissue. Figure 2C shows the subject's baseline PET-MRI fusion image.

Figure 2D shows axial sections of the ETA T1-MRI scan. Tracer uptake on the ETA PET image (Fig. 2E) is observed to correspond to the site of the GBM on the subject's ETA MRI as seen in the PET-MRI fusion image (Fig. 2F). Compared to baseline, the ETA PET scan showed reduced ^18^F-ML-10 uptake in the center of the tumor region, which previously demonstrated greatest uptake. Moreover, compared to baseline, new regions of ^18^F-ML-10 uptake were observed at the tumor periphery.

To further investigate changes in ^18^F-ML-10 uptake distribution between the baseline (Fig. 2B and G) and ETA (Fig. 2E and H) time points, a subtraction cluster analysis was performed and fused to the subject's baseline T1-MRI scan (Fig. 2I) using MIM 5.4 image analysis software. The subtraction cluster analysis calculates *z*-scores of the fractional changes in normalized tracer uptake with respect to baseline on a voxel-by-voxel basis. To be considered significant, a voxel *z*-score must exceed a threshold value. Additionally, the voxel must be part of a cluster whose minimum size corresponds to a threshold *P*-value. The *P*-value/cluster-size correspondence is deduced from Gaussian random field theory given the PET scan resolution (6 mm). In this analysis, a voxel *z*-score threshold of 3 and a cluster *P*-value threshold of 0.05 were used. The results of the subtraction cluster analysis highlight visible changes in ^18^F-ML-10 uptake pattern before and after therapy initiation. Regions of the GBM exhibiting high baseline ^18^F-ML-10 uptake show reduced uptake at ETA (blue), while new regions (compared to baseline) of ^18^F-ML-10 uptake are observed at the tumor periphery (red/orange).

## Discussion

This case report presents the first reported use of PET with ^18^F-labeled ML-10 to evaluate changes in apoptosis in GBM before and early after therapy. Both the baseline and ETA ^18^F-ML-10 PET scans showed tracer uptake that corresponded to the GBM anatomical location on the associated MRI scans (Fig. 2), with low tracer uptake in other areas of normal brain. Moreover, after RT+temozolomide therapy, the ETA ^18^F-ML-10 PET scan showed a different pattern of tracer distribution compared to baseline.

Some ^18^F-ML-10 accumulation is observed in the scalp and calvarium at both imaging time points (Fig. 2), anatomically corresponding to the location of the stereotactic surgery incision, and is observed to increase between the two imaging time points. A possible explanation of this extraneous uptake is apoptosis due to traumatic cell injury. This explanation is supported by previous results from fluorescence imaging studies using didansyl cystine (DDC), an apoptosis probe with similar functional characteristics as ML-10 (Reshef et al. 2008).

Molecular imaging of apoptosis using ^18^F-ML-10 PET is a promising new method for evaluation of therapy response in GBM. After 3 weeks of therapy, changing patterns of ^18^F-ML-10 uptake between baseline and ETA were visible.
